# First graduate of the Master of Nursing in Advanced Practice in Oncology in Chile

**DOI:** 10.1590/0034-7167.202376suppl401

**Published:** 2023-07-31

**Authors:** Pilar Alejandra Espinoza Quiroz, Carolina Andrea González Araya, Elsa Ana Cabrera Acosta

**Affiliations:** I Universidad San Sebastián, Faculdade de Medicina e Ciências. Santiago, Chile; II Clinical Trial Unit, Instituto Oncológico, Fundación Arturo López Pérez. Santiago, Chile; III Hospital Metropolitano de Santiago y Clínica Dávila. Santiago, Chile

In 2020, the guidelines for Advanced Nursing Practice (APN) implementation^(1)^ were published by the International Council of Nursing (ICN). On that occasion, I was invited to comment on the reality of specialist nurses in Chile, expressing the expectation of implementing an APN program, specifically Clinical Nurse Specialists (CNS). CNS are one of the most internationally recognized APN functions, and its development has followed the traditional scope of nursing practice, but taking it to a higher degree, the master’s degree, where, through academic rigor, scientific reasoning and critical thinking, it promotes optimal, safe and quality health care^(1)^.

In 2021, I had the honor of participating in a REBEn editorial that sought to promote the APN implementation in Latin America and the Caribbean^(2)^, and today we have the opportunity to share the graduation of the first oncology CNS (OCNS), which graduated in a master’s program developed in Chile with international support, including the main author of the ICN paper Madrean Schober, Susan Kelly-Weeder, Associate Dean of Graduate Programs at Boston College and Executive Director at the Sylvester Comprehensive Cancer Center, all from the United States. Furthermore, the program has a significant number of oncologists from multiple specialties who share their experiences and knowledge with students, graduating part of the program’s academic core.

To finish the OCNS program (2 years), it is necessary to carry out a clinical internship that allows demonstrating professionals’ skills in a health organization, in addition to the development of a continuous quality improvement project in the context of health as an academic activity. The first graduation project carried out was entitled “Implementation of a navigation pilot project in patients of an oncological hospital in Chile”, seeking to establish the role of oncology nurse navigators with a group of patients with breast cancer. The results showed that the implementation of paper reduced access times to different stages of the oncological process by an average of 33.4% (10% to 43.5%), in which participating patients highlighted the importance of having a nursing professional who follows, supports and educates during the different stages of their illness, in addition to emotionally and psychologically supporting them and their families.

Currently, OCNS have training that allows them to make complex decisions in the specialty area, optimizing nursing care through a systemic approach that involves direct and indirect care. Direct care includes patients and their family and includes, for instance, a holistic approach during patient assessment, diagnosis, treatment, follow-up and education, promoting and facilitating patient access to the interdisciplinary team, supporting them in decision-making, defending their rights and incorporating ethics into their care. Indirect care, in turn, involves clinical network management of the different levels of cancer care and their relationship with the community. In this context, OCNS promote interprofessional collaborative team work, acting as a leader, mentor and nursing professor to promote continuous professional development, guiding the implementation of continuous quality improvement projects, integrating the best scientific evidence available in clinical practice and facilitating change and innovation in organizations, with a focus on quality and cost-effectiveness of care.

The path to obtaining the graduation of the first student meant overcoming several implementation challenges, such as the need to have multispecialty oncologists and chemical pharmacists with expertise in oncology, willing to share their knowledge and experiences with students, explain to health professionals OCNS’ role and competencies and how it differs from other APN roles, in order to facilitate their insertion in the clinic, socialize APN formation’s progress with legislators to simultaneously advance in recognizing their leading role in the country. On the positive side, this experience has allowed us to meet several health professionals who recognize the need to train OCNS and have provided invaluable support for this educational project, carrying out an e-learning program, in addition to the practice, allowing us to have students from all over Chile who believed in this project and agreed to be the pioneers in the insertion of role in our health system.

The challenge that OCNS must face now is the insertion in their health organizations in their new function, where the closing activities of a master’s program (clinical practice and graduation project) are expected to facilitate the socialization of this new role within their organizations with health teams and with their patients. Considering master’s training and the disciplinary context in Chile, it is important to demonstrate their new professional skills in areas with limited organizational development at the local level, such as the network oncology process management through the different levels of care, adding to the community the implementation of a model that incorporates the best evidence available in nursing care, in addition to promoting the subject’s professional development by leading education and mentoring projects that meet patients’ and organization’s needs.
