# ERRATUM

**DOI:** 10.1590/0034-7167.20237605e04

**Published:** 2023-11-10

In the article “First graduate of the Master of Nursing in Advanced Practice in Oncology in Chile”, with DOI number: https://doi.org/10.1590/0034-7167.202376suppl401, published in Revista Brasileira de Enfermagem, 2023(Suppl 4):e76suppl401, in the second paragraph:

Where it read:

In 2021, I had the honor of participating in a REBEn editorial that sought to promote the APN implementation in Latin America and the Caribbean^(^2^)^, and today we have the opportunity to share the graduation of the first oncology CNS (OCNS), which graduated in a master’s program developed in Chile with international support, including the main author of the ICN paper Madrean Schober, Susan Kelly-Weeder, Associate Dean of Graduate Programs at Boston College and Executive Director at the Sylvester Comprehensive Cancer Center, all from the United States.

It reads:

In 2021, I had the honor of participating in a REBEn editorial that sought to promote the APN implementation in Latin America and the Caribbean^(^2^)^, and today we have the opportunity to share the graduation of the first oncology CNS (OCNS), which graduated in a master’s program developed in Chile with international support, including the main author of the ICN paper Madrean Schober, Susan Kelly-Weeder, Associate Dean of Graduate Programs at Boston College and Deborah Anne Piehl, Executive Director at the Sylvester Comprehensive Cancer Center, all from the United States.

